# Lipid Storage and Interferon Response Determine the Phenotype of Ground Glass Hepatocytes in Mice and Humans

**DOI:** 10.1016/j.jcmgh.2021.03.009

**Published:** 2021-03-23

**Authors:** Yuri Churin, Karuna Irungbam, Christoph S. Imiela, David Schwarz, Hans-Joachim Mollenkopf, Uta Drebber, Margarete Odenthal, Oleg Pak, Magdalena Huber, Dieter Glebe, Martin Roderfeld, Elke Roeb

**Affiliations:** 1 Department of Gastroenterology, Justus Liebig University, Giessen, Germany; 2 Institute for Veterinary Food Science, Faculty of Veterinary Medicine, Justus Liebig University, Giessen, Germany; 3 Core Facility Microarray, Max Planck Institute for Infection Biology, Berlin, Germany; 4 Institute for Pathology, University Hospital of Cologne, Cologne, Germany; 5 Center for Molecular Medicine, University of Cologne, Cologne, Germany; 6 Excellence Cluster Cardiopulmonary System, University of Giessen and Marburg Lung Center, Justus Liebig University, Giessen, Germany; 7 Institute for Medical Microbiology and Hospital Hygiene, University of Marburg, Marburg, Germany; 8 Institute of Medical Virology, National Reference Centre for Hepatitis B and D Viruses, Justus Liebig University Giessen, Giessen, Germany

**Keywords:** Hepatitis B, GGH, Surface Proteins, Intracellular Aggregates, ApoB, apolipoprotein B, CHB, chronic hepatitis B, ER, endoplasmic reticulum, FIT, fat storage–inducing transmembrane protein, GGH, ground glass hepatocyte, HBs, hepatitis B virus surface protein, HBsAg, hepatitis B surface antigen, HBV, hepatitis B virus, IRF, interferon regulatory factor, ISG, interferon stimulated gene, LD, lipid droplet, LHBs, large hepatitis B virus surface protein, PLIN2, perilipin 2, TG, triacylglycerol

## Abstract

**Background and Aims:**

A histopathological hallmark of chronic hepatitis B virus (HBV) infection is the presence of ground glass hepatocytes (GGHs). GGHs are liver cells that exhibit eosinophilic, granular, glassy cytoplasm in light microscopy and are characterized by accumulation of HBV surface (HBs) proteins in the endoplasmic reticulum (ER). More important, GGHs have been accepted as a precursor of HCC and may represent preneoplastic lesions of the liver.

**Methods:**

Here we show that the reason for ground glass phenotype of hepatocytes in patients with chronic hepatitis B (CHB) and in HBs transgenic mice is a complex formation between HBs proteins and lipid droplets (LDs) within the ER.

**Results:**

As fat is a main component of LDs their presence reduces the protein density of HBs aggregates. Therefore, they adsorb less amount of eosin during hematoxylin-eosin staining and appear dull in light microscopy. However, after induction of interferon response in the liver LDs were not only co-localized with HBs but also distributed throughout the cytoplasm of hepatocytes. The uniform distribution of LDs weakens the contrast between HBs aggregates and the rest of the cytoplasm and complicates the identification of GGHs. Suppression of interferon response restored the ground glass phenotype of hepatocytes.

**Conclusions:**

Complex formation between HBs and LDs represents a very important feature of CHB that could affect LDs functions in hepatocytes. The strain specific activation of the interferon response in the liver of HBs/c mice prevented the development of GGHs. Thus, manipulation of LDs could provide a new treatment strategy in the prevention of liver cancer.

## Graphical abstract

**Figure:**
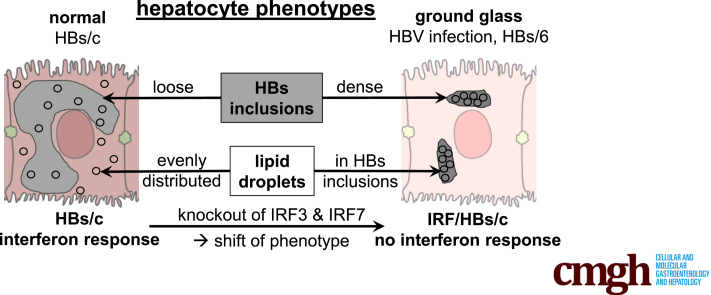

Chronic infection with hepatitis B virus (HBV) affects 350–400 million individuals worldwide and is the leading cause of liver cirrhosis and hepatocellular carcinoma worldwide.^1^ HBV is one of the smallest enveloped DNA viruses and the prototype member of the family of *Hepadnaviridae*. The HBV genome contains 4 overlapping open-reading frames that encode the viral polymerase, HBV surface proteins (HBs), the structural core protein and the nonstructural precore protein, also known as secreted e-antigen, and the X protein.^2^ HBs—the large HBs (LHBs), middle HBs, and small HBs—can be distinguished by their different domains and glycosylation status. The carboxyterminal domain containing small HBs is present in all surface proteins, preS1 N-terminal extension only in LHBs, and preS2 in LHBs and middle HBs.^3^ These 3 forms of HBs represent hepatitis B surface antigen (HBsAg).^4^

A histopathological hallmark of chronic HBV infection is the presence of ground glass hepatocytes (GGHs).^5^, ^6^, ^7^ GGHs are liver cells that have eosinophilic granular and glassy cytoplasm on light microscopy.^8^ The GGHs are different in morphology and distribution at different stages of chronic HBV infection.^9^, ^10^, ^11^ Two major types of GGHs exist. Type I GGHs usually are distributed sporadically in liver lobules and occur throughout the replicative phases. Normally, they have slightly eccentric nuclei with an accumulation of ground glass substances in the cytoplasm.^5^^,^^6^^,^^9^^,^^11^ Type II GGHs usually appear at late nonreplicative stages or in cirrhotic liver and are distributed in large clusters with a marginal expression of HBsAg.^9^^,^^12^ Furthermore, it was shown that type I GGHs harbored mutants with deletions in preS1 region, whereas type II GGHs contained mutants with deletions in the preS2 region that defines a cytotoxic T lymphocyte immune epitope, and may represent an immune escape mutant.^13^^,^^14^ More important, preS mutants, especially preS2, induce DNA damage as well as multiple intracellular signaling pathways that provoke hepatocarcinogenesis. Thus, GGHs may represent the preneoplastic lesions of HBV-related hepatocellular carcinoma.^14^, ^15^, ^16^, ^17^, ^18^, ^19^, ^20^, ^21^

The endoplasmic reticulum (ER) membrane is the site of lipid droplets (LDs) formation, storage organelles having unique structure including a hydrophobic core of neutral lipids (sterol esters and triacylglycerols [TGs]), which is covered by a phospholipid monolayer that is decorated by specific proteins.^22^ One of the most abundant LD proteins belong to the perilipin family (perilipin 1–5) and protect LDs from lipase action.^23^ Perilipin 2 (PLIN2) is the major hepatic LD protein.^24^ PLIN3 was recently found to regulate the hepatic LD biogenesis, and cellular levels of PLIN2 and PLIN3 correlate with TG storage level.^25^^,^^26^

In this study, we demonstrate that the reason for the ground glass phenotype of hepatocytes in the liver of patients with chronic hepatitis B (CHB) and HBs transgenic mice is a complex formation between HBs and LDs in the ER. Complex formation resulted in the arrest of LDs in the ER and led to the appearance of GGHs. Induction of interferon response eliminated and suppression of interferon response restored the ground glass phenotype of hepatocytes.

## Results

### Ground Glass Phenotype of Hepatocytes in the Liver of HBs Transgenic Mice Is Associated With Intracellular Lipid Storage

**Figure 1:**
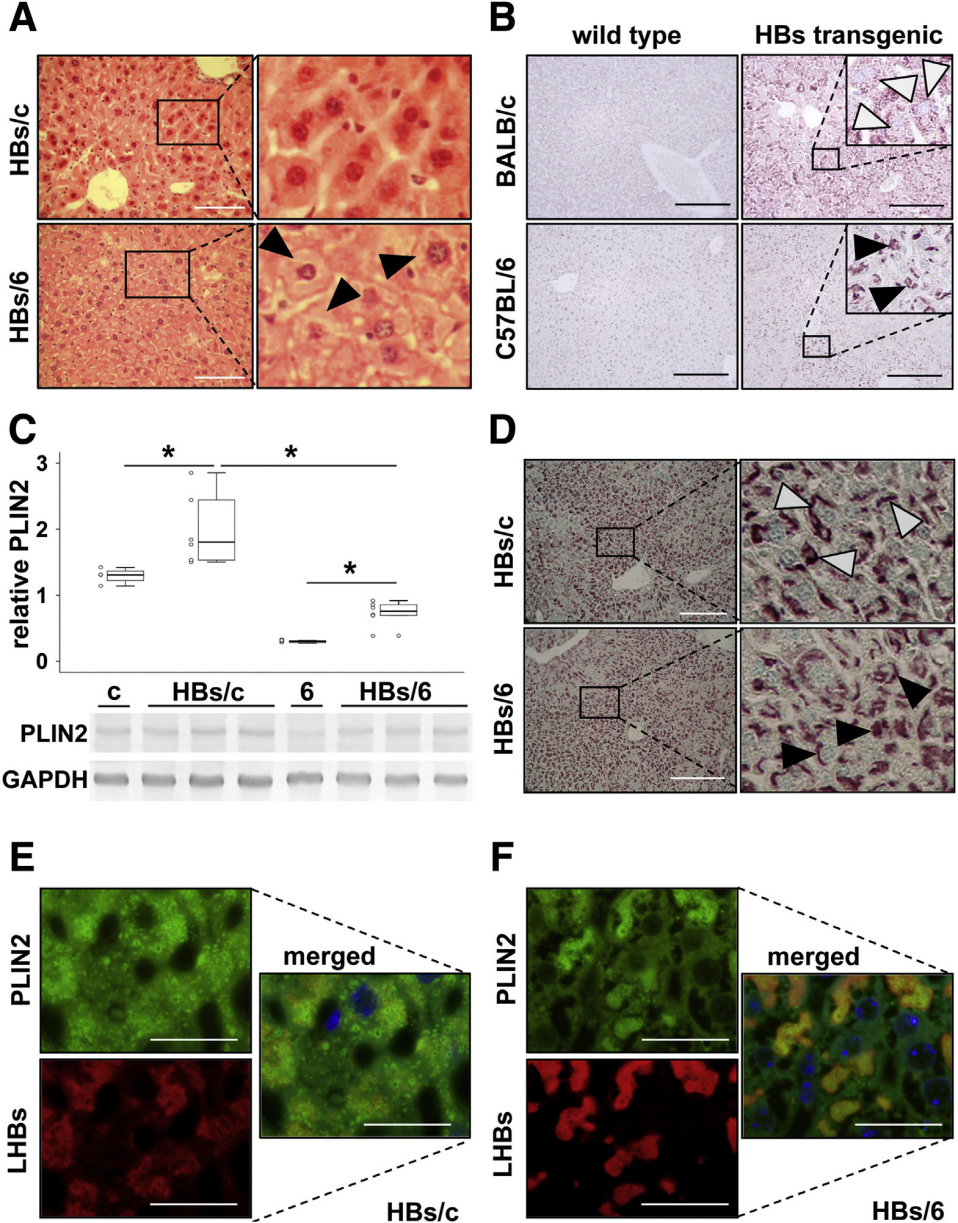
**The presence of GGHs in the liver of HBs transgenic mice depends on genetic background.** (*A*) Hematoxylin and eosin staining of paraffin liver sections of HBs transgenic mice on BALB/c (*upper panel*) and C57BL/6 (*lower panel*) genetic background revealed the presence of GGHs in HBs/6 mice. Arrowheads indicate typical GGHs, which mainly occur on C57BL/6 background. *Scale bars* = 100 μm. (*B*) Immunohistochemical analyses of paraffin liver sections of BALB/c (*upper panel*) and C57BL/6 (*lower panel*) wild-type (*left panels*) mice. Liver sections of HBs transgenic mice were shown on the right side. HBs/c and HBs/6 were performed using specific antibodies against constitutive LD protein PLIN2. *White arrowheads* indicate that PLIN2-positive LDs were distributed evenly in the cytoplasm; *black arrowheads* indicate dense accumulation of PLIN2-positive LDs. *Scale bars* = 200 μm. (*C*) Western blot analysis of total protein lysates from the liver of HBs/c and HBs/6 using anti-PLIN2 specific antibodies. Equal protein loading was confirmed with anti-GAPDH antibodies. c indicates total protein lysate from the liver of BALB/c wild-type mouse; 6 indicates total protein lysate from the liver of C57BL/6 wild-type mouse. Densitometric analysis was performed with ImageJ software. Mann-Whitney *U* test was applied to test significance. ^∗^*P* < .05. n = 4–6 mice per group. These are representative immunoblotting data of 3 independent experiments. (*D*) Immunohistochemical analyses of LHBs in HBs/c and HBs/6 mice. *White arrowheads* indicate HBs distributed evenly in the cytoplasm; *black arrowheads* indicate dense accumulation of HBs. *Scale bars* = 200 μm. Immunofluorescence analyses of paraffin liver sections of (*E*) HBs/c and (*F*) HBs/6 were performed using anti-PLIN2 (*green*) and anti-LHB (*red*) antibodies. Nuclei were stained with Hoechst 33342 (*blue*). Colocalization of these 2 proteins appears in *yellow* (merge). *Scale bars* = 25 μm. Inserts show enlarged images representing the outlined area. n = 5–10 mice per group.

Hepatocytes from HBs transgenic mice on C57BL/6 genetic background (HBs/6) display the characteristic features of ground glass cells^27^ and resemble type II GGHs.^13^ The histochemical analysis of livers from HBs transgenic mice on BALB/c genetic background (HBs/c)^28^ revealed only very low numbers of GGHs (Figure 1*A*, upper panels). We confirmed the existence of many GGHs in HBs/6 (Figure 1*A*, lower panels, black arrowheads). HBs is co-translationally integrated into the ER membrane.^2^ The ER membrane is also the site of LDs formation, and expression levels of the major LD protein in hepatocytes, PLIN2, correlate with the storage level of TGs.^26^ To test whether HBs expression influences LD formation, we performed immunohistochemical and western blot analyses of PLIN2 in the livers of HBs/c and HBs/6 mice. HBs expression resulted in stronger accumulation of PLIN2 in hepatocytes of transgenic compared with corresponding wild-type mice (Figure 1*B* and *C*). More interesting, this analysis revealed a strong difference of PLIN2 distribution in hepatocytes of HBs/6 (Figure 1*B*, black arrowheads) compared with HBs/c (Figure 1*B*, white arrowheads). The hepatic PLIN2 expression pattern in HBs/6 mice was very similar to the expression pattern of HBs (Figure 1*D*, black arrowheads), whereas in hepatocytes of HBs/c mice, PLIN2 was distributed more evenly (Figure 1*B*, white arrowheads). Immunofluorescence analyses demonstrated PLIN2 and HBs colocalization in hepatocytes of both transgenic mice strains (Figure 1*E* and *F*). HBs inclusions contain lots of small LDs in hepatocytes of HBs/6 mice, whereas we could not detect LDs in other parts of the hepatocytic cytoplasm (Figure 1*F*). In the case of HBs/c mice, we observed apparently less colocalization of PLIN2 and HBs. Moreover, PLIN2 was detected additionally in the whole cytoplasm of hepatocytes (Figure 1*E*). Thus, the presence of GGHs in the liver of HBs transgenic mice was associated with intracellular lipid storage and depended on the genetic background.

**Figure 2:**
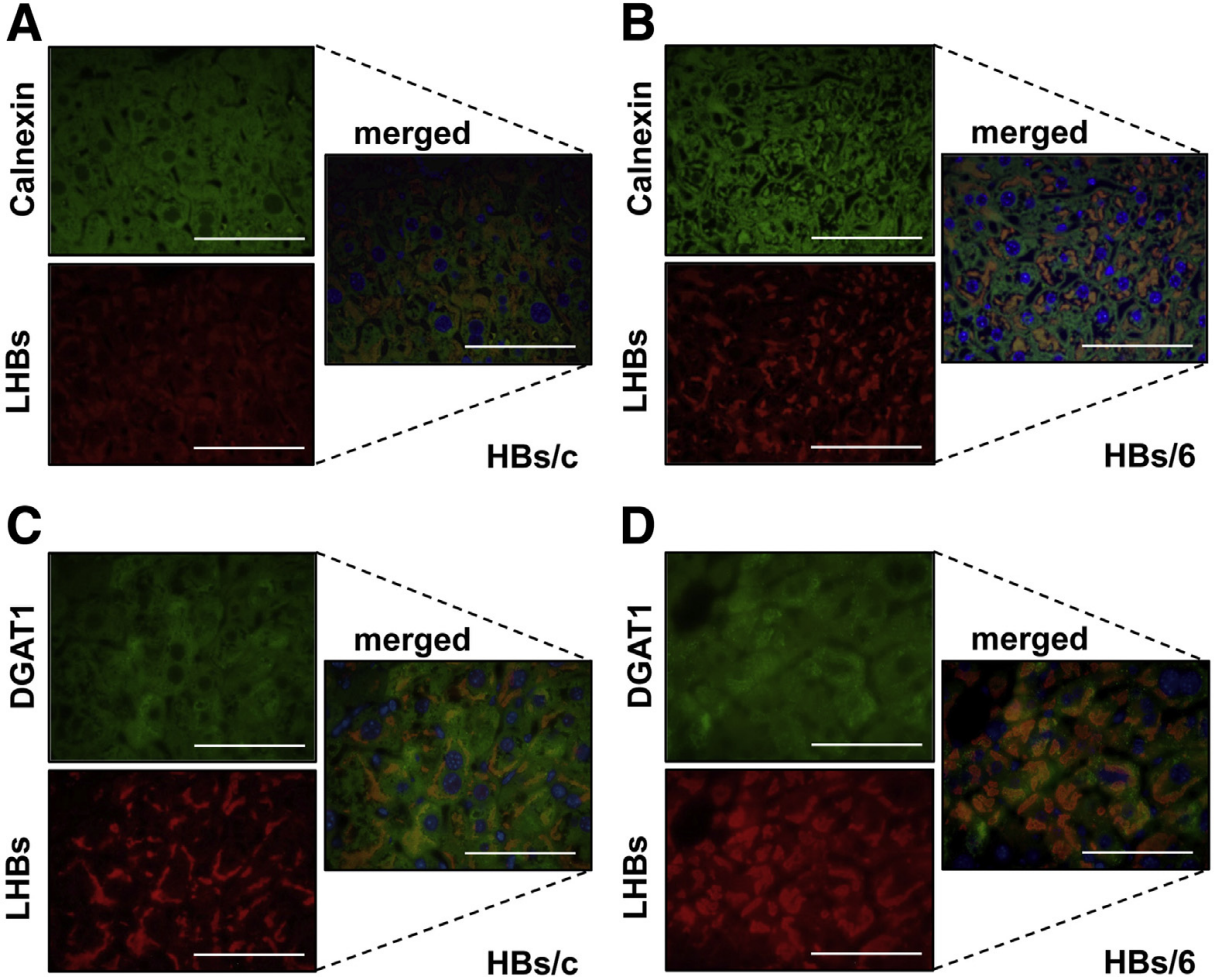
**HBs are localized in ER.** Immunofluorescence analysis of paraffin liver sections from (*A*, *C*) HBs/c mice and (*B*, *D*) HBs/6 mice. (*A*, *B*) Staining was performed using anti-Calnexin (*green*) and anti-LHBs (*red*) antibodies. Nuclei were stained with Hoechst 33342 (*blue*). Colocalization (merged) of these 2 proteins appears in *yellow*. (*C*, *D*) Staining was performed using anti-DGAT1 (*green*) and anti-LHBs (*red*) antibodies. Nuclei were stained with Hoechst 33342 (*blue*). Colocalization (merged) of these 2 proteins appears in *yellow*. *Scale bars* = 25 mm.

Expression of HBs in mouse liver resulted in the accumulation of these proteins in the ER.^27^ In the liver of HBs/c and HBs/6 mice, we observed a colocalization of HBs with the integral ER proteins calnexin (Figure 2*A* and *B*) and DGAT1 (diacylglycerol-O-acyltransferase 1) (Figure 2*C* and *D*). Thus, HBs accumulated in the ER and colocalized with the LDs constitutive protein PLIN2 indicating that LDs were arrested completely in the hepatocytic ER of HBs/6 and partially in the ER of HBs/c mice.

### LDs Colocalize With HBs Aggregates in Hepatocytes of Patients With CHB

**Figure 3:**
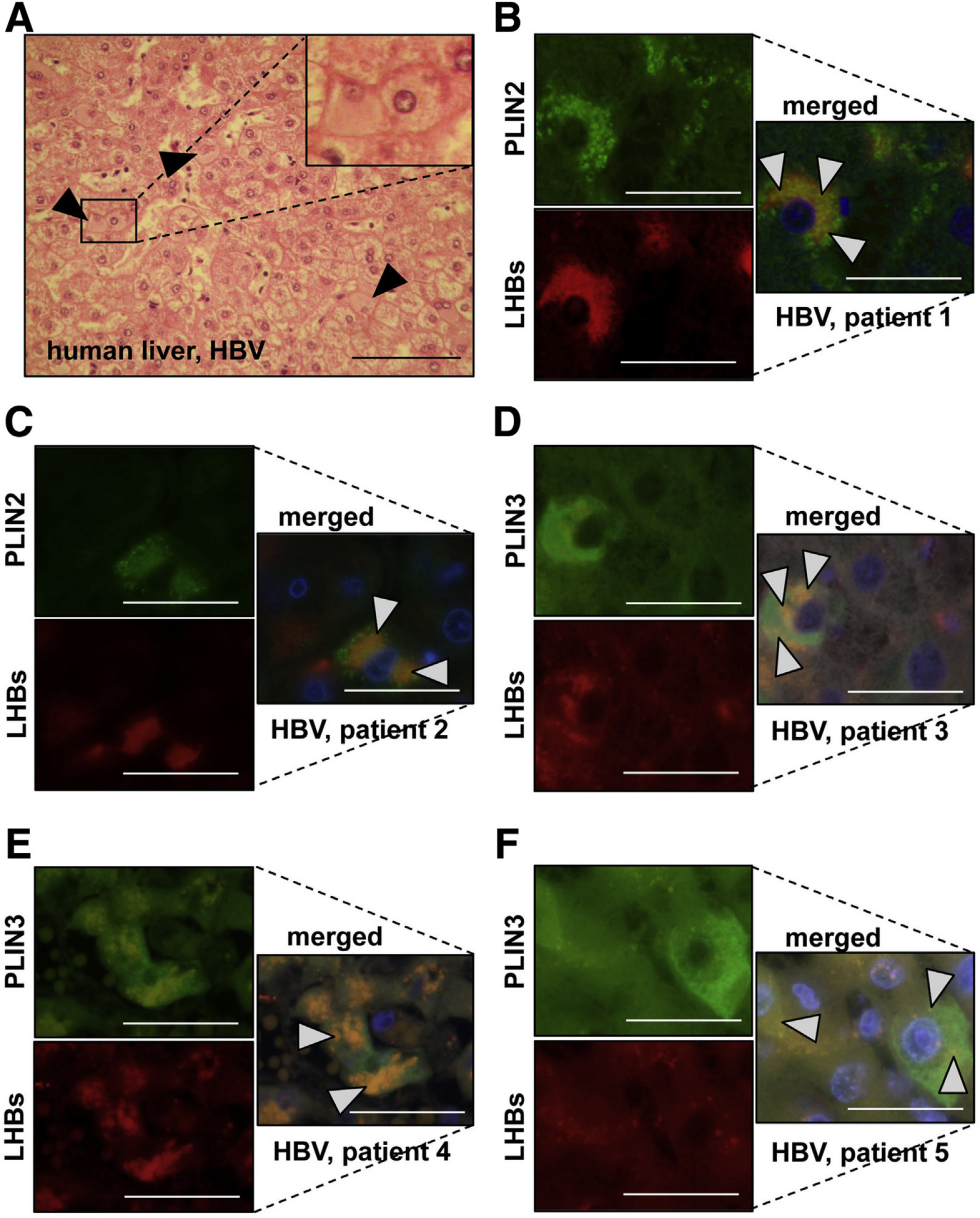
**Ground glass material in the liver of patients with CHB infection constitutes a complex of HBs and LDs.** (*A*) Hematoxylin and eosin staining of paraffin liver sections from a patient with CHB infection revealed the presence of GGHs. The *black arrowheads* indicate typical GGHs. *Scale bar* = 100 μm. (*B–F*) Immunofluorescence analysis of paraffin liver sections from 5 different patients with CHB infection was performed using anti-PLIN2 (*green*) and anti-LHB (*red*) antibodies (*lower panels*). Nuclei were stained with Hoechst 33342 (*blue*). Colocalization (merged) of these 2 proteins appears in *yellow* (*white arrowheads*). *Scale bar* = 25 μm. Representative microphotographs are shown.

Histological analysis of the liver from patients with CHB revealed the presence of GGHs (Figure 3*A*). Interestingly, immunofluorescence staining using anti-PLIN2, anti-PLIN3, and anti-LHBs specific antibodies demonstrated a colocalization of these proteins in the hepatocytes of these CHB patients (Figure 3*B–F*). Analysis of enlarged images of human and mouse samples (Figures 1*E* and *F* and 3*B–F*) revealed that HBs aggregates are filled with LDs as inclusions.

### Interferon Response Affects the Development of Ground Glass Phenotype of Hepatocytes

**Figure 4:**
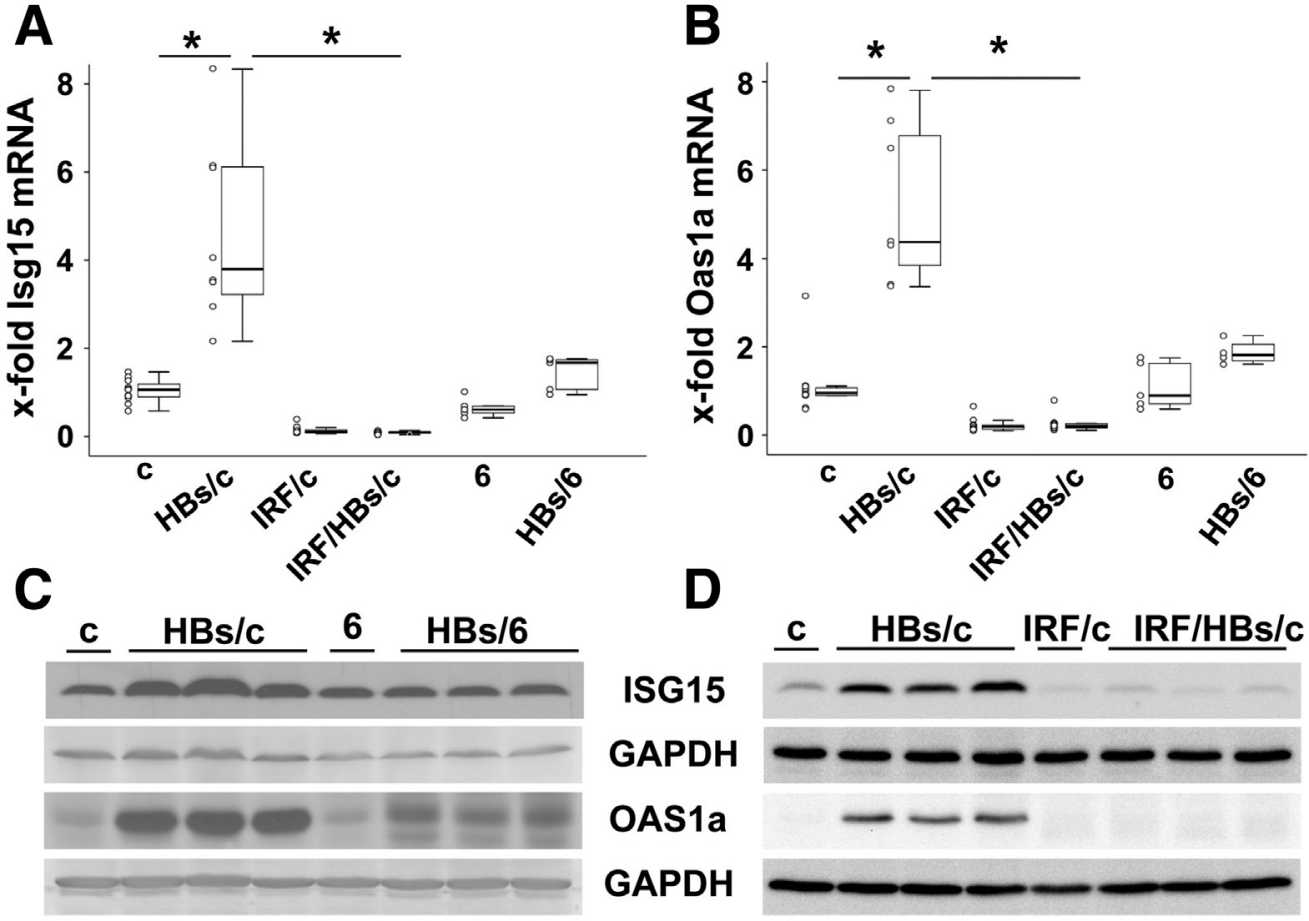
**Inactivation of interferon response reduced ISG15 and OAS1a expression.** Transcriptional analysis of ISG15 (*A*) and OAS1 (*B*) Mann-Whitney *U* test was applied to test significance. ^∗^*P* < .05. n = 5–13 mice per group. Western blot analyses of total protein lysates from the livers of (*C*) HBs/c and HBs/6 and (*D*) from HBs/c, IRF/c, and IRF/HBs/c mice were performed using anti-ISG15 and anti-OAS1a specific antibodies. Equal protein loading was confirmed with anti-GAPDH antibodies. c indicates total protein lysate from the liver of BALB/c wild-type mouse; 6 indicates total protein lysate from the liver of C57BL/6 wild-type mouse; IRF/c indicates total protein lysates from the liver of IRF3/7 double-knockout mice. n = 5–13 mice per group. These are representative immunoblotting data of 3 independent experiments.

Microarray analysis of total RNA from the livers of HBs/c and HBs/6 mice revealed a strong activation of interferon-stimulated genes (ISGs) expression in HBs/c but not in HBs/6 mice^29^ (Table 1). The results from the microarrays were validated by quantitative real time PCR for the ubiquitin-like protein Isg15^30^ and Oas1a (2'-5' oligoadenylate synthetase 1a)^31^ (Figure 4*A* and *B*).Western blot analysis of total liver lysates confirmed these findings on the protein level. The expression of ISG15^30^ and OAS1a^31^ was more pronounced in the liver of HBs/c in comparison with HBs/6 mice (Figure 4*C* and *D*). Taken together, these data strongly suggest that HBs expression in the liver of transgenic mice activated an interferon response in BALB/c mice only.

**Table 1:** Interferon Regulated Genes Are Upregulated in the Liver of HBs Transgenic Mice on BALB/c Genetic Background

Accession number	Sequence name	Annotation	HBs/c		HBs/6	
			Fold change	*P* value	Fold change	*P* value
NM_010738	Ly6a	Lymphocyte antigen 6 complex, locus A	9.39	2.96 × 10^–32^	1.10	.09892
NM_008331	Ifit1	Interferon-induced protein with tetratricopeptide repeats 1	7.88	9.50 × 10^–36^	1.03	.80399
NM_145153	Oas1f	2'-5' oligoadenylate synthetase 1F	6.34	0	1.29	.01331
NM_133871	Ifi44	Interferon-induced protein 44	5.73	1.67 × 10^–31^	1.33	.00151
NM_011909	Usp18	Ubiquitin specific peptidase 18	5.57	0	1.64	2.82 × 10^–20^
NM_145211	Oas1a	2'-5' oligoadenylate synthetase 1A	4.43	1.51 × 10^–21^	1.34	.03228
NM_016850	Irf7	Interferon regulatory factor 7	4.20	0	1.24	.0957
NM_010501	Ifit3	Interferon-induced protein with tetratricopeptide repeats 3	3.85	0	1.34	.0002
NM_015783	Isg15	ISG15 ubiquitin-like modifier	3.84	0	1.46	.00081
NM_145209	Oasl1	2'-5'-oligoadenylate synthetase-like 1	3.69	1.51 × 10^–15^	1.30	.06098
NM_021384	Rsad2	Radical S-adenosyl methionine domain containing 2	3.39	0	1.29	.12543
NM_029000	Gvin1	GTPase, very large interferon inducible 1	3.19	0	1.65	7.16 × 10^–11^

HBs, hepatitis B virus surface protein.

### Inactivation of Interferon Response Restores GGH Development in the Liver of HBs/c Mice

In order to investigate whether an interferon response in HBs/c mice is responsible for the phenotypic differences between HBs/c and HBs/6 mouse strains, we prepared triple-transgenic mice by crossing HBs/c with the interferon regulatory factor (RF) 3 and IRF7-double-deficient mice.^32^ IRFs play a central role in the induction of type I IFN production.^33^^,^^34^ As IRF3 and IRF7 were globally knocked out, we intended to inhibit interferon response in the liver by inactivation of these IRFs. Quantitative reverse-transcription polymerase chain reaction (Figure 4*A* and *B*) and Western blot analysis (Figure 4*D*) of total protein lysates from the livers of HBs/c, IRF3^–/–^/IRF7^–/–^ (IRF/c), and HBs triple-transgenic mice (IRF/HBs/c) demonstrated that the inactivation of the transcription factors IRF3 and 7 resulted in a suppression of interferon response in HBs/c mice. Thus, the interferon response activation by HBs in the liver of transgenic mice on BALB/c genetic background was dependent on the transcription factors IRF3 and IRF7.

**Figure 5:**
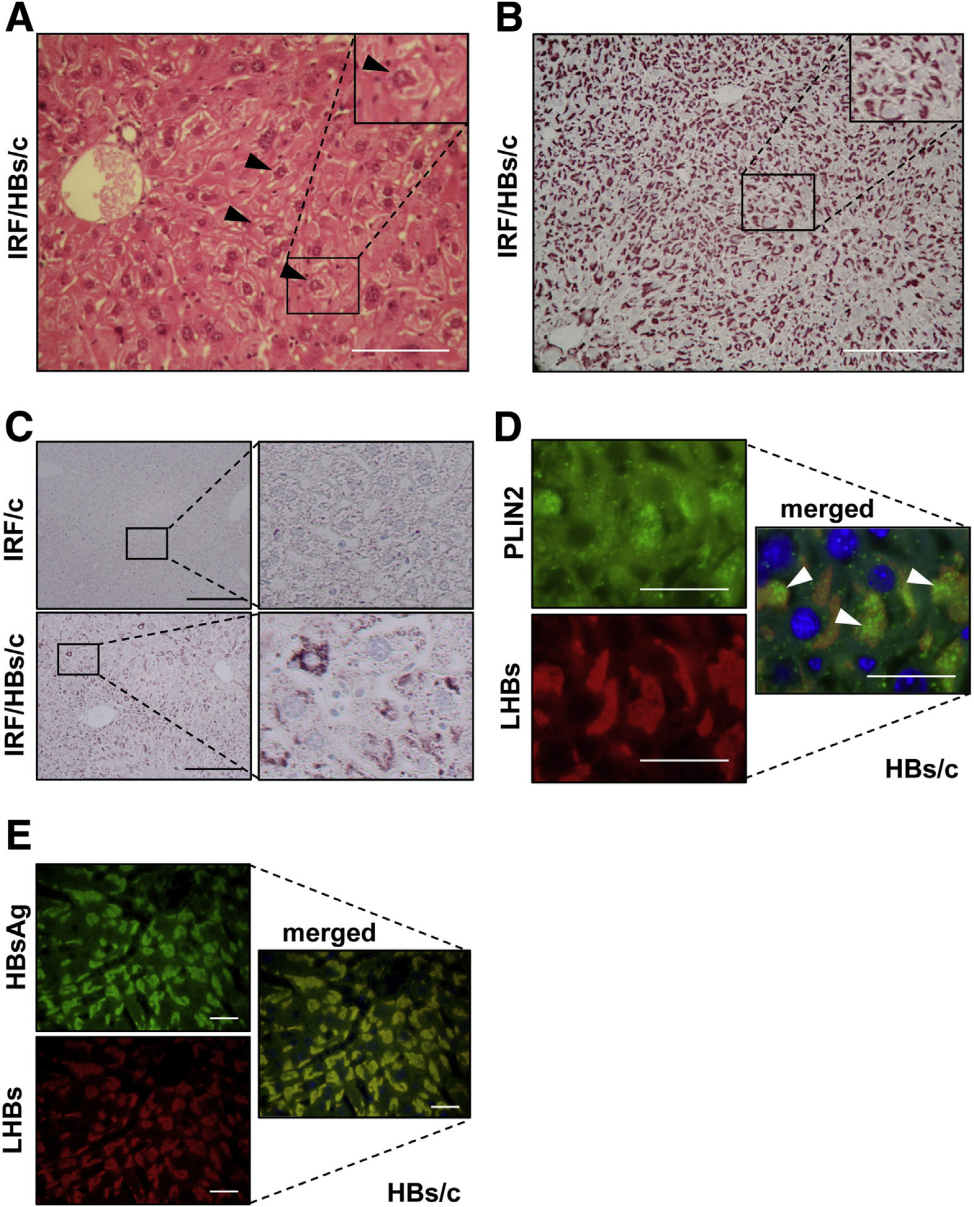
**Inactivation of interferon response restores GGHs development in the liver of HBs/c mice.** (*A*) Hematoxylin and eosin staining of paraffin-embedded liver sections from IRF/HBs triple-transgenic mice revealed the presence of GGHs. The *black arrowheads* indicate typical GGHs. *Scale bar* = 100 μm. Immunohistochemical analyses of paraffin-embedded liver sections of IRF/HBs triple-transgenic mice were performed using (*B*) specific antibodies against LHBs and (*C*) specific antibodies against constitutive LDs protein PLIN2. *Scale bars* = 200 μm. (*D*) Immunofluorescence analyses of paraffin-embedded liver sections were performed using anti-PLIN2 (*green*) and anti-LHBs (*red*) antibodies. Nuclei were stained with Hoechst 33342 (*blue*). Colocalization of these 2 proteins appears in *yellow*. (*E*) Distributions of LHBs and HBsAg in the liver HBs transgenic mice are completely matched. Representative immunofluorescence analysis of HBs/c mouse liver using anti-HBsAg (*green*) and anti-LHBs (*red*) antibodies. Nuclei were stained with Hoechst 33342 (*blue*). Colocalization of these 2 proteins appears in *yellow* (overlay). *Scale bars* 25 μm. n = 5–13 mice per group.

Histochemical analyses of liver sections from IRF/HBs/c mice demonstrated the presence of GGHs (Figure 5*A*). Furthermore, the suppression of the interferon response in HBs/c mice led to a PLIN2 and LHBs expression pattern that was similar to HBs/6 mice (Figure 5*B* and *C*). Moreover, in IRF/HBs/c, LDs containing PLIN2 were barely detectable outside of HBs aggregates (Figure 5*D*). Taken together, the strain specific activation of the interferon response in the liver of HBs/c mice prevented the development of GGHs.

## Discussion

**Figure 6:**
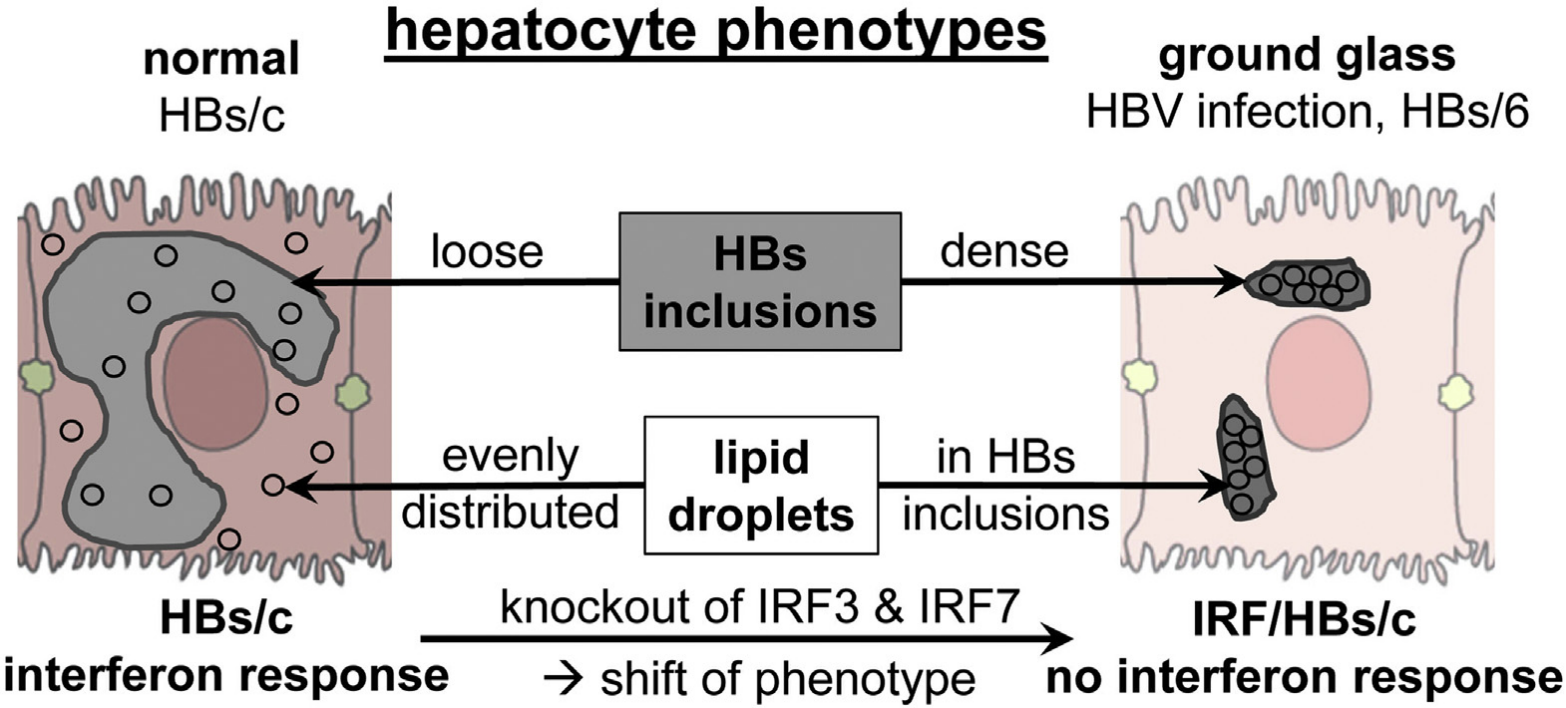
**Schematic summary of the results from the current study.** HBs aggregates are filled with LDs as inclusions in the hepatocytes of patients with CHB and of HBs/6 transgenic mice (right). Hepatocytes of HBs/c mice accumulated LDs also in the cytoplasm, which weakened the contrast between HBs aggregates and the rest of the cytoplasm and complicated the identification of GGHs (left). This effect was abolished by inactivation of the interferon response, which suggests that one of the ISG protein products facilitated the change of the phenotype (ie, budding of LDs from ER and transport into the cytoplasm).

The reasons for the appearance of GGHs are poorly understood. Here, we show that HBs aggregates are filled with LDs as inclusions in the hepatocytes of a patient with CHB and of HBs transgenic mice. Figure 6 summarizes the main findings of the current study schematically.

Fat as the major content of LDs is dissolved during the preparation of samples for histological analysis and HBs aggregates resembled some kind of sponge. Hence, we suggest a “sponge” model of ground glass material. In this case, LDs represent the pores of the “sponge” filled with fat. Porous material has a lower density. Therefore, it adsorbed less amount of eosin during hematoxylin and eosin staining and appeared dull in light microscopy. Hepatocytes of HBs/c mice accumulated LDs also in the cytoplasm, which weakened the contrast between HBs aggregates and the rest of the cytoplasm and complicated the identification of GGHs.

After formation in the ER membranes, LDs bud from the ER and are localized in the cytoplasm.^22^ Two groups of proteins play important roles in LD biogenesis: seipins and fat storage–inducing transmembrane (FIT) proteins.^35^ Mammalian cells possess 2 FIT proteins, FIT1, which is muscle specific, and FIT2, which is expressed in most other tissues.^36^ In cells lacking FIT2 proteins, most LDs are embedded in the ER membrane and exposed to the ER lumen.^37^ The interference of HBs with the function of FIT2 in the ER membrane might be a possible reason for LD arrest in the ER. Furthermore, it has been shown previously that apolipoprotein B (ApoB), a major component of very low-density lipoproteins, is deposited in a region around LDs called the ApoB crescent. Abnormally lipidated ApoB binds tightly to the ER membranes and arrests any LDs departure from the ER.^38^ Accumulation of HBs in ER might affect ApoB lipidation and disturb LD budding.

Although we still have to figure out the reason for induction of interferon response in the liver of HBs/c mice, LDs were visualized in hepatocytes of these mice not only colocalized with HBs in the ER, but also in the rest of the cytoplasm. As this effect was abolished by inactivation of the interferon response, we assume that one of the ISG protein products^29^ facilitated the change of the phenotype (ie, budding of LDs from ER and transport into the cytoplasm). This effect could prevent the formation of GGHs.

Interestingly, we observed the induction of interferon response in the liver of transgenic HBs/c mice and to a certain degree in transgenic HBs/6 mice (Figure 4), although HBV wild-type infection does not readily induce host cellular interferon response in chimpanzees^39^ and humans.^40^ In accordance to our findings, interferon response was demonstrated in human liver–derived cells in vitro with very high-level HBV expression such as HepaRG cells.^41^ HBs transgenic mice do reflect specific features of chronic HBV infection with very high-level HBV expression (ie, massive accumulation of intracellular HBs, fibrogenesis, and carcinogenesis).^28^^,^^42^ Therefore, our results also suggest that the hepatocellular accumulation of high amounts of HBs may promote an interferon response under certain genetic conditions that involve the acquired immune status (BALB/c-Th2 prone > C57BL/6-Th1 prone). This observation might have clinical relevance to overcome the malignant effects of GGH formation (eg, by pharmacologic modulation of T helper cell signaling).

LDs play a very important role in host-pathogen interactions.^43^^,^^44^ One of the best characterized pathogens with regard to the interaction with LDs is the hepatitis C virus.^45^, ^46^, ^47^ However, little is known about the interaction of HBV and LDs.^48^^,^^49^ Our present study demonstrates that LDs play a role at least at the late stage of CHB, being responsible for the formation of the hepatocellular ground glass phenotype. Furthermore, we have previously shown that male HBs/c develop less tumors than male HBs/6 mice,^28^ which might suggest the importance of ground glass material formation for precancerous lesions in hepatocytes. In addition to the main function of LDs as cytoplasmic organelles that store neutral lipids and their importance for energy metabolism, LDs play an essential role in vitamin storage and signaling precursors, in managing cell stress, and in protein maturation, storage, and turnover.^50^ Sequestration of LDs in HBs aggregates could impair some of these important functions and thus contribute to the development of hepatocellular cancer.

## Materials and Methods

### Human Subjects

The human tissue specimens were collected in the Institute for Pathology, University Clinic of Cologne, Germany (Biomasota 13-091), and the use was approved by the Ethics Commission of the University of Cologne (Az. 18-052).

### Mouse Strains

Transgenic mice were maintained at the Central Animal Laboratory of the Justus Liebig University Giessen, Germany, under specified pathogen-free conditions. This study was carried out in strict accordance with the recommendations in the Guide for the Care and Use of Laboratory Animals of the German law of animal welfare. Mice received humane care, and all experiments were approved by the Committee on the ethics of Animal Experiments of the Regierungspräsidium Giessen, Giessen, Germany (permit number: V54-19c 20 15 h 01 GI20/10 No. 128/2014). All efforts were made to minimize suffering.

Generation and characteristics of transgenic lineages Tg(Alb-1HBV), internal designation HBs/6, on C57BL/6 genetic background has been described previously.^42^ These mice were crossed back to BALB/c genetic background for at least 8 generations. The transgenic mouse strain obtained was internally designed HBs/c.

An IRF3 and IRF7 double-deficient mouse strain on C57BL/6 genetic background was kindly provided by Prof. A. Krug (Technical University Munich, Munich, Germany) and has been described previously.^32^ These mice were crossed back to BALB/c genetic background for at least 8 generations. The obtained transgenic mouse strains were internally designed IRF for IRF3^–/–^/IRF7^–/–^ double-transgenic mice and IRF/HBs for triple-transgenic mice. At the age of 12 weeks, male mice were sacrificed and liver samples were collected and preserved for analyses in accordance with the further application.

### Histology

For histology liver samples were fixed in 4% neutral buffered paraformaldehyde (#2213.3; Carl Roth, Karlsruhe, Germany) at 4°C for 16 hours and embedded in paraffin. Paraffin-embedded liver samples were cut into 3- to 5-μm sections, and routine hematoxylin and eosin was performed as described previously.^28^

Immunohistochemical and immunofluorescence analyses were performed using 3-μm paraffin sections. The samples were boiled for 10 minutes in citrate buffer (pH 6.0). To perform immunohistochemical stainings, peroxidase activity was blocked with 3% hydrogen peroxide (#8070.1; Carl Roth). Sections were then blocked with 10% bovine serum albumin (PAA, Pasching, Austria) and 2.5% normal horse serum (#MP-7401; Vector Laboratories, Burlingame, CA) and incubated with specific antibodies according to the manufacturer`s protocols. Immunohistochemical analyses were performed using ImmPRESS Peroxidase Detection Reagents (#MP-7401, MP-7402; Vector Laboratories). Color reaction was developed with VECTOR VIP Peroxidase Substrate Kit (#SK-4100, Vector Laboratories). To perform immunofluorescence analyses, the pretreated sections were blocked with 10% bovine serum albumin (#BSA-1T; PAA) for 1 hour and incubated overnight with specific antibodies at 4°C. Secondary goat anti-rabbit Alexa488 (#A11008), goat anti-mouse Alexa546 (#A11030)–conjugated antibodies (Molecular Probes, Eugene, OR), and goat anti-guinea pig FITC (#90101; Progen, Heidelberg, Germany). Photographs were taken using a Leica DMRB microscope (Leica, Wetzlar, Germany) equipped with a Canon EOS 600D with Canon EOS Utility 2 software, version 2.14 (Canon, Tokyo, Japan).

Immunofluorescence analyses of HBs transgenic mouse liver using anti-LHBs (MA18/7 detects an epitope [DPXF] in the preS1 amino acids 20–23 (31–34 in genotype A])^51^ and anti-HBsAg (#20-HR20; Fitzgerald, North Acton, MA) antibodies demonstrated a complete match of staining (Figure 5*E*).

### Western Blot Analysis

Total protein lysates were prepared from crushed liver tissue in 1× Laemmli buffer. After boiling for 10 minutes, the samples were subjected to sodium dodecyl sulfate polyacrylamide gel electrophoresis and transferred to polyvinylidene difluoride membranes (#IPCH00010; Merck, Darmstadt, Germany). Visualization of proteins was performed by horseradish peroxidase–linked antibodies and the ECL Chemiluminescence Detection Kit (#34087, Pierce ECL; Thermo Fisher Scientific, Dreieich, Germany) according to the manufacturer’s protocols. Densitometric quantification was performed using ImageJ software (v.1.52a, National Institutes of Health, Bethesda, MD).

### Antibodies

ADRP/Perilipin 2 (#15294-1-AP), DGAT1 rabbit polyclonal antibodies (#11561-1-AP) and GAPDH (glyceraldehyde-3-phosphate dehydrogenase) mouse monoclonal antibodies (#60004-1-Ig; ProteinTech Group, Chicago, IL), Calnexin rabbit polyclonal antibodies (#ADI-SPA-865; Enzo Life Sciences, Lörrach, Germany), HBsAg (20-HR20) rabbit polyclonal antibodies (#20-HR20; Fitzgerald Industries, Acton, MA), PLIN3 guinea pig polyclonal antibodies (#GP30s; Progen, Heidelberg, Germany), ISG15 (#sc-166755; Santa Cruz Biotechnology, Heidelberg, Germany), and Oas1a mouse monoclonal antibodies (#sc-365072; Santa Cruz Biotechnology) were used according to the manufacturer’s protocols. Anti-LHBs mouse monoclonal antibodies (Virology, Giessen, Germany) were described previously.^52^

### Microarray Analysis

Microarray experiments were performed with total RNA from the liver of 12-week-old mice as described previously.^53^ Microarray experiments were performed as dual-color hybridizations. Total RNA was isolated using TRIzol (#15596018; Life Technologies, Carlsbad, CA). Quality control and quantification of the total RNA amount was assessed using an Agilent 2100 Bioanalyzer (#G2939BA; Agilent Technologies, Waldbronn, Germany) and a NanoDrop 1000 Spectrophotometer (Thermo Fisher Scientific, Langenselbold, Germany). RNA labeling was performed with the Low RNA Input Fluorescent Linear Amplification Kit (#5185-5818; Agilent Technologies). In brief, messenger RNA was reverse-transcribed and amplified using an oligo-dT-T7-promotor primer (New England Biolabs, Frankfurt am Main, Germany), and resulting complementary RNA was labeled either with Cyanine 3-CTP or Cyanine 5-CTP (both from New England Biolabs). After precipitation, purification, and quantification, 1.25 μg of each labeled complementary RNA was fragmented and hybridized to whole mouse genome 44k microarrays, according to the supplier’s protocol (#G4170-90012; Agilent Technologies). Scanning of microarrays was performed with 5-μm resolution, using a DNA microarray laser scanner (Agilent Technologies). Raw microarray image data were analyzed with the Image Analysis/Feature Extraction software G2567AA (version A.9.5.1; Agilent Technologies). The extracted MAGE-ML files were analyzed with the Rosetta Resolver Biosoftware, Build 6.1 (Rosetta Biosoftware, Seattle, WA). Ratio profiles comprising single hybridizations were combined in an error-weighted fashion to create ratio experiments. A 1.5-fold change expression cutoff for ratio experiments was applied together with anticorrelation of ratio profiles, rendering the microarray analysis highly significant (*P* < .01), robust, and reproducible. The data presented here have been deposited in National Center for Biotechnology Information’s Gene Expression Omnibus and are accessible through Gene Expression Omnibus Series accession number GSE40826.

## Statistical Analysis

Statistical analysis was performed with SPSS version 26.0 software (IBM Corporation, Armonk, NY). Man-Whitney *U* tests were applied in order to define differences between expression levels. Relative expression is shown in box-and-whisker plots. The upper and lower hinges of the box represent the 75th and 25th percentile, respectively. The line indicates the median value; error bars represent the minimum and maximum. Additionally, all individual data points are depicted. Significant differences are pointed out (∗*P* < .05).

All authors had access to the study data and had reviewed and approved the final manuscript.
